# How the incentive to contribute affects contributions in the one-shot public goods game

**DOI:** 10.1038/s41598-020-75729-8

**Published:** 2020-10-30

**Authors:** Pieter van den Berg, Peter Dewitte, Ine Aertgeerts, Tom Wenseleers

**Affiliations:** Lab of Socioecology and Social Evolution, KU Leuven, Naamsestraat 59, 3000 Leuven, Belgium

**Keywords:** Social evolution, Human behaviour

## Abstract

Enmeshed in various social structures, humans must often weigh their own interest against the interest of others—including the common interest of groups they belong to. The Public Goods Game (PGG), which succinctly pits individual interest against group interest, has been a staple of research into how people make such decisions. It has been studied in many variations, in the laboratory and (increasingly) online. One of the defining parameters of the PGG is the marginal per capita return of the group project (MPCR), which determines the incentive for contributing to the group project relative to the incentive of keeping points in the personal account. The effect of MPCR on contributions has been investigated before, but its effects have never been characterised with high resolution. Here, we present a systematic and high-resolution investigation of the effect of MPCR in groups of three. We do this in a large-scale online decision making experiment recruiting participants from Amazon Mechanical Turk. Our results provide a fine-grained account of the relationship between incentive to cooperate on the one hand and cooperation on the other, and can help to provide a basis for choosing MPCR magnitudes for future research endeavours using online PGG studies.

## Introduction

Human social decision making often involves weighing one’s own interests against the interests of others. Since humans are embedded in many social structures, such decisions are ubiquitous in people’s lives, and have accordingly been studied across scientific disciplines for decades^1–3^. Many experimental methods have been developed to study how humans navigate such decisions, the Public Goods Game (PGG) paramount among them. In the PGG, participants are divided in groups, and have to simultaneously decide how much of an endowment to allocate to a group project. Next, the total amount invested in the group project is multiplied by a factor larger than one (and smaller than the group size), and the resulting sum is subsequently distributed equally among all group members. If played only once (the ‘one-shot’ version of the game), this set-up ensures that individuals maximise their earnings by investing nothing in the group project (i.e., investing nothing is the dominant strategy), whereas the total group earnings are maximised if all group members invest their entire endowment.

Many variations of the PGG—both one-shot and repeated—have been studied over the decades. The repeated (or iterated) PGG confronts participants with several subsequent PGGs in the same group, so that investing in the group project can increase one’s earnings in the longer term (through eliciting higher contributions from other group members in later rounds). Examples of much-studied extensions include the addition of a punishment phase after the investment phase^4,5^, in which group members can pay to harm the earnings of fellow group members, and designs in which group members receive different endowments (to study the effects of inequality)^6,7^. There have also been investigations into the effects of the multiplication factor and the group size on cooperation, which together determine the marginal per capita returns (MPCR) of investing in the group project (higher multiplication factors increase MPCR, whereas larger group sizes decrease MPCR). Overall, the conclusions of these studies have been that the MPCR matters—higher MPCR tends to lead to higher contributions, especially when group size is not too large^8–12^. However, these studies typically include only two or three different values for MPCR, and are therefore not able to make detailed quantitative predictions about its effect on contributions in the PGG.

More detailed knowledge about the effect of MPCR in PGGs is desirable for at least two reasons. First, it can give us more insight in the shape of the relationship between MPCR and cooperation—studies with only a few values for MPCR only give us a very course idea about this. Second, it can be instrumental for researchers in choosing the magnitude of MPCR for future PGG studies. This is especially the case for studies that make use of more than one value for MPCR, such as studies that are interested in within- or between-group heterogeneity in MPCR^13–15^. A detailed picture of the relationship between MPCR and cooperation can make sure that researchers developing such studies choose MPCR from a range in which changes in MPCR actually matter for cooperation (i.e., they are not chosen from a ‘plateau’ in which cooperation rates do not respond to changes in MPCR).

In this study, we systematically investigate the effect of MPCR on contributions in one-shot PGGs in groups of three (i.e., by changing the multiplication factor). Through the online labour market Amazon Mechanical Turk, we were able to obtain relatively large sample sizes for twelve different MPCR values. We show that contributions increase in response to increasing MPCR, and that this increase eventually levels off at a maximum average contribution. Based on our results, we identify the range of MPCR that is associated with the largest response in contributions.

## Methods

Our experiment included a total of 648 participants recruited through Amazon Mechanical Turk, a large online labour market. All recruited participants were based in the USA and were over 18 years of age (min age = 18, mean age = 36.0, max age = 73; 57.1% male, 42.4% female, 0.5% other/would rather not say). After recruitment on Amazon Mechanical Turk, individuals clicked a link that led them to our interactive experimental platform (programmed in LIONESS Lab^16^). There, they received detailed instructions, answered a quiz to test their comprehension of the experimental set-up (see Supplementary Materials for details), and played a test trial of two rounds to let them get used to the experimental environment without consequences for their earnings. After this, the actual experimental session would begin.

We ran a total of 12 treatments in which individuals played ten subsequent one-shot Public Goods Games in groups of three. Each treatment had a different multiplication factor that stayed constant over all rounds: 1.1–2.0 in steps of 0.1, 2.2 and 2.5, corresponding to MPCRs of 0.367–0.667 in steps of 0.033, 0.733 and 0.833. All these MPCRs are in the range between 1/N (0.333) and 1, so in all cases the PGG reflects an actual social dilemma in which the dominant strategy is not to contribute any points to the group project (i.e., this is individually most profitable), but in which contributing does increase the total group earnings (the range of MPCR considered in our study is roughly the same range as would be achieved by fixing the multiplication factor at 2.5 but varying group size between 3 and 7). Each treatment included 54 participants and was run in three batches with 18 participants each (in total, our data set contains 5,921 decisions that were made in time—see more on time limits below). Individuals were made aware that they were interacting with new interaction partners every round both in the introduction and at the start of each round. Individuals were made explicitly aware of the multiplication factor, and were presented with a (graphical) representation of the public goods dynamics with the multiplication factor of their treatment in the introduction (see Supplementary Materials for example screens).

After the test trial, individuals were grouped with two other participants and given an endowment of 5 points (in the rest of the manuscript, we rescale contributions so that the maximum contribution is equal to 1). In the ‘Decision screen’, all group members simultaneously decided how much of their endowment to contribute to the group project. Individuals had to make this decision within 20 s (in the first two rounds, the time limit was 35 s)—if they did not decide within the time limit, they automatically made a random contribution (this happened in 1.4% of cases). Next, on the ‘Results screen’, they were shown how much all group members had contributed, the total number of points that were present in the group project both before and after multiplication with the multiplication factor, their share of the group project, and their total earnings for that round (points not invested in the group project + share of the group project). Because some drop-out inevitably occurs, it occasionally happened that some participants could not be connected to two interaction partners. In such cases (which constituted 7% of all situations), participants were shown a special screen that informed them that they could not make a decision and would automatically earn 5 points in that round (2.5% of participants faced this situation in more than three out of the ten rounds). After ten rounds, participants filled out a brief questionnaire eliciting some basic demographic information, and were subsequently informed of their total earnings. They were paid through Amazon Mechanical Turk. All participants received a ‘flat fee’ of $1.50, and an extra payment that was proportional to the number of points accumulated during the experiment (50 points = $1). In total, participants earned on average $2.87 (min earnings = $2.04, max earnings = $3.93). Sessions lasted around 15 min. The experimental setup was approved by the Social and Societal Ethics Committee of KU Leuven (SMEC), and all methods were performed in accordance with the relevant guidelines and regulations. Informed consent was obtained from all participants.

We constructed two general linear models to predict contributions: one that took all rounds into account, and one for the first round only. The former was a mixed-effects linear model that included the following fixed predictors: (1) a natural cubic spline in function of MPCR, (2) a natural cubic spline in function of the round number, (3) a natural cubic spline in function of the interaction of MPCR and round number. This model included individual nested in experimental session as a random intercept. Each of the spline predictors had two degrees of freedom (we systematically checked different degrees of freedom for each variable separately and ended up with this model based on AIC). The latter model was a linear model that included only a natural cubic spline in function of MPCR with two degrees of freedom as a predictor (again, the degrees of freedom were based on AIC) and did not include any random effects (this yielded a better AIC than a model that included session as a random effect; individual was not included because this model only included a single data point for each individual). See Supplementary Materials for more details on both these models.

## Results

**Figure 1:**
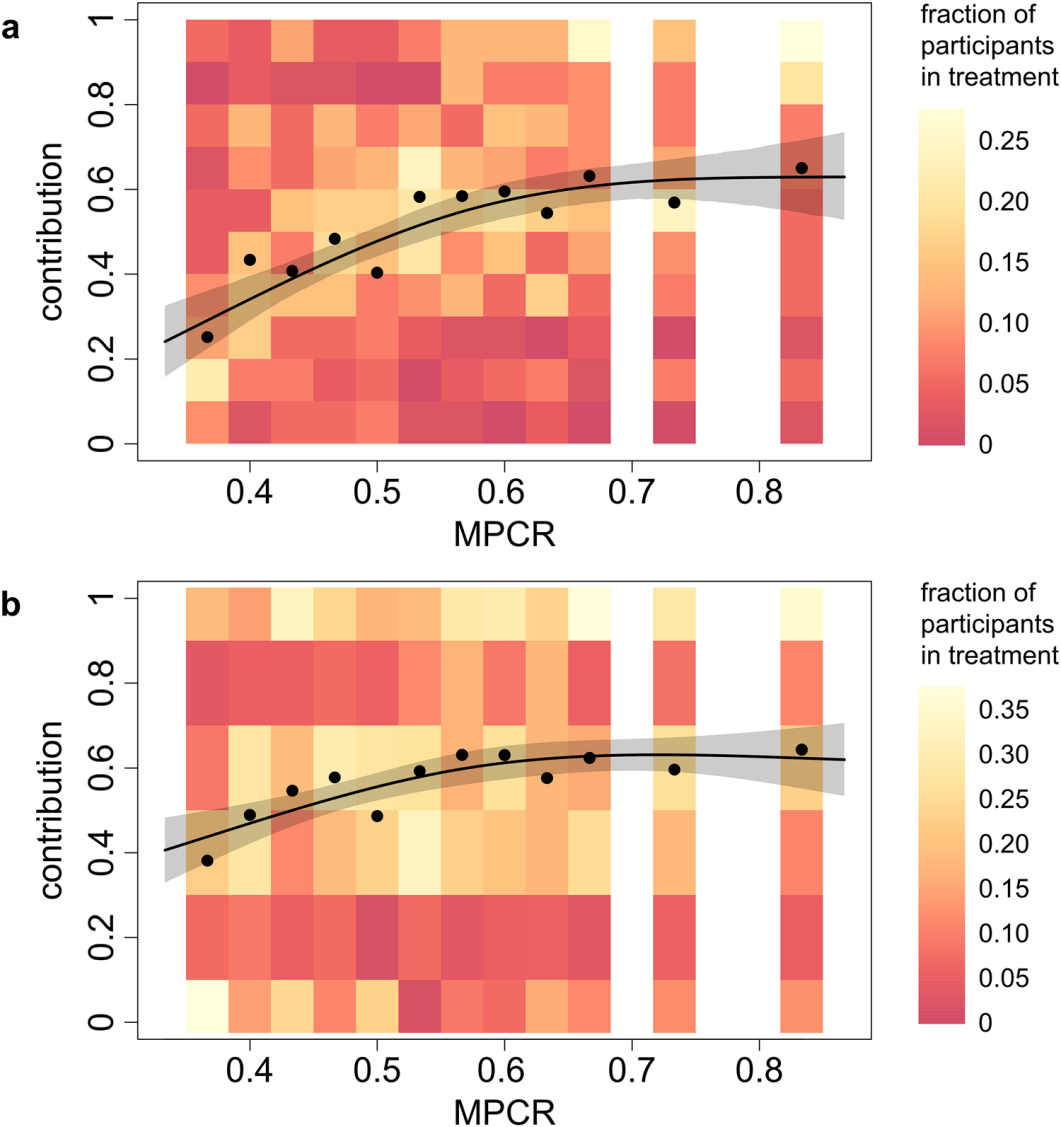
Contributions in the Public Goods Game depending on marginal per capita returns of investing in the group project (MPCR). **(a)** Average contributions over all ten rounds (every round was ‘one-shot’, i.e. participants interacted with new interaction partners every round), **(b)** Contribution in the first round only (in this round, there is no possibility that participants’ decisions were influenced by earlier rounds). Coloured rectangles indicate the fraction of individuals with an average contribution falling in the indicated range for each treatment (see legend). Black dots show average contribution rates for each treatment. The line shows the cooperation response to multiplication factor predicted by our models based on the data (for figure **a**, based on period 6; see Supplementary Materials for details of the statistical models). The shading around the line indicates the 95% confidence interval of the mean.

Figure 1a gives a detailed overview of how MPCR affects contributions over all rounds, and how our statistical model infers this relationship from the data. There is a substantial increase in average contribution between MPCR of 0.367 and 0.55, after which the increase decelerates. Upwards of MPCR of about 0.7, further increases in MPCR hardly lead to an increase in contribution anymore. Specifically, our model predicts that the average contribution increases by between 4 and 12% for each 0.02 increase in MPCR for in the range 0.35–0.55, by between 1 and 4% for MPCR in the range 0.55–0.7, and by less than 1% upwards of 0.7. The maximum contribution level that was reached lies around 0.63; well below the maximum possible contribution. Overall, our model predicts that 80% of the variability in contribution response to MPCR occurs in the range of MPRC under 0.58, and that 90% lies in the range under 0.64.

Despite the fact that our participants played one-shot games (i.e., interacted with different interaction partners each round), we find a temporal trend: a decrease in contributions over the rounds, especially in treatments with relatively low MPCR (see Supplementary Materials for details). This suggests that participants may have been influenced in their decisions by what happened in past interactions—even if they would not interact with these same interaction partners again. To exclude any such effects, we also analysed how MPCR affected contributions in the first round only. Since there is no history before the first round, this can be considered as a ‘cleaner’ measure of behaviour in the one-shot game. Although less pronounced, we still observe the same pattern for first-round decisions only: contributions increase with MPCR when MPCR is relatively low, and level off for higher MPCR levels (see Fig. 1b). Similar to the model for all rounds, our statistical model that only includes first round data predicts that 80% of the variability in contribution response to MPCR occurs in the range of MPRC under 0.57, and that 90% lies in the range under 0.63.

The temporal decline in contributions over rounds was more pronounced for low MPCR than for high MPCR. Specifically, in the treatment with lowest MPCR (0.367), average contributions declined from 0.38 to 0.17, whereas contributions only declined from 0.68 to 0.61 in the treatment with highest MPCR (0.833). See Supplementary Materials for effect plots that visualise this pattern, as well as plots of raw data over time for each treatment. It is not straightforward to ascertain what causes these differences, but they may be associated with differences in history (i.e., contributions of interaction partners in previous rounds; see Supplementary Materials for an exploration of this effect).

## Discussion

Our results show that average contributions increase as a response to increasing MPRC, relatively steeply for low MPCR, then decelerating and finally levelling off around a maximum average contribution. Most of the variation in contributions in response to MPCR occurs for MPCR under 0.58 (80% of the variation) or 0.64 (90% of the variation). We also observed that the maximum average contribution level is not equal (or even very close) to the maximum possible contribution—even in treatments where the return on investing in the group project is quite high (in our case, MPCR = 0.833).

Our results can be useful for calibrating MPCR in future PGG studies. This is especially the case for studies that include more than one value of MPCR, such as studies that are interested in within- or between-group variation in MPCR or in the response to changing MPCR over time. For example, for a study in which one would like to study how variation in MPRC over time affects cooperative behaviour, one would want to select values of MPCR from a range that are actually expected to elicit variation in contributions. Our results provide a basis for choosing appropriate values of MPCR for such studies.

Our study was conducted within groups of three. Extrapolating our results to different group sizes should be done with care, although there are some reasons to suggest that our results can (at least qualitatively) be informative for other group sizes. There is no strong evidence that group size per se (while holding MPCR constant) has a strong effect on contributions—results on this have been mixed, pointing to a relatively weak positive effect of group size at best (as long as group size is not very large). Isaac et al.^8^ found that contribution rates were similar for groups of 4 and 10 participants and also for even larger groups if MPCR was high, but, for relatively low MPCR, contribution rates were significantly higher in larger groups (40 or 100 participants). The latter result has recently been replicated for one-shot PGGs as well^17^. In a meta-analysis^9^, Zelmer did not find a significant effect of group size per se on contributions in PGGs. Nosenzo et al.^10^ found that the effect of group size was dependent on MPCR (contributions increase with group size for low MPCR but decreased for high MPCR), whereas Lugovskyy et al.^11^ found a weak support for a positive effect of group size (increasing from two to four) on contributions in some of their studied conditions. Weimann et al.^18^ found almost no group size effect (but did find an MPCR effect) in a study with very large groups (60 or 100) and very low MPCR (0.02 or 0.04). Overall, it is difficult to draw straightforward conclusions about the effect of group size on contributions, but, especially as long as group sizes are not too large, there is little evidence that group size has a strong effect. Hence, overall, we would argue that our results also have some merit for other group sizes, if only for giving an initial indication of which values of MPCR are most interesting to consider. Of course, detailed results for different group sizes are needed to have more reliable estimates for the impact of the MPCR on contributions.

We ran our study through the online labour market Amazon Mechanical Turk. Earlier work has shown that participants from this platform tend to contribute more in PGGs than participants in the lab, but that their behaviour is qualitatively very similar^19^. Because of this quantitative difference, it is not immediately straightforward to apply our results to laboratory situations. For example, it is unclear whether the maximum contribution level (the level at which contributions no longer increase due to increases in MPCR) will be the same on MTurk as in the lab. It is conceivable that the contribution response to MPCR in the lab only starts levelling off at higher values of MPCR (because contributions are lower overall). This is a question that needs to be addressed empirically.

## Supplementary information

Supplementary Information.

## Data Availability

All data analysed in the current study, the R codes that were used to run our statistical analyses, and the LIONESS code used to run our experiment are available in an Open Science Framework repository (doi:10.17605/osf.io/mtgyf).
