# Supplementary material for: How the incentive to contribute affects contributions in the one-shot public goods game
Source: Sci Rep. 2020 Oct 30;10:18732. doi: 10.1038/s41598-020-75729-8 (PMC7599224; doi:10.1038/s41598-020-75729-8)
Supplement: Supplementary file 1 — Supplementary Information. [file 41598_2020_75729_MOESM1_ESM.pdf]

# Supplementary Materials

accompanying the manuscript

'How the incentive to contribute affects contributions in the one-shot Public Goods Game'

by Pieter van den Berg, Peter Dewitte, Ine Aertgeerts and Tom Wenseleers

**CONTENTS**


1. Example screens of experimental software *page 2*
2. Comprehension questions to test participants *page 8*
3. Details of statistical models *page 10*
4. Raw data of contributions over time for each treatment *page 12*
5. The effects of history *page 13*

# 1. Example screens of experimental software

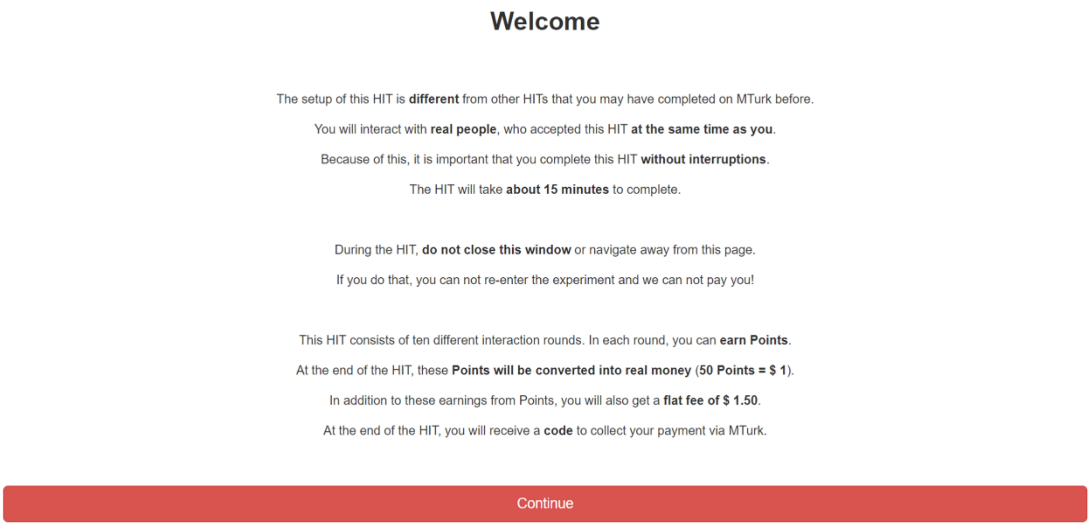


**Fig S1. Welcome screen.** This is the first screen the participants saw after entering our experiment from Amazon Mechanical Turk.

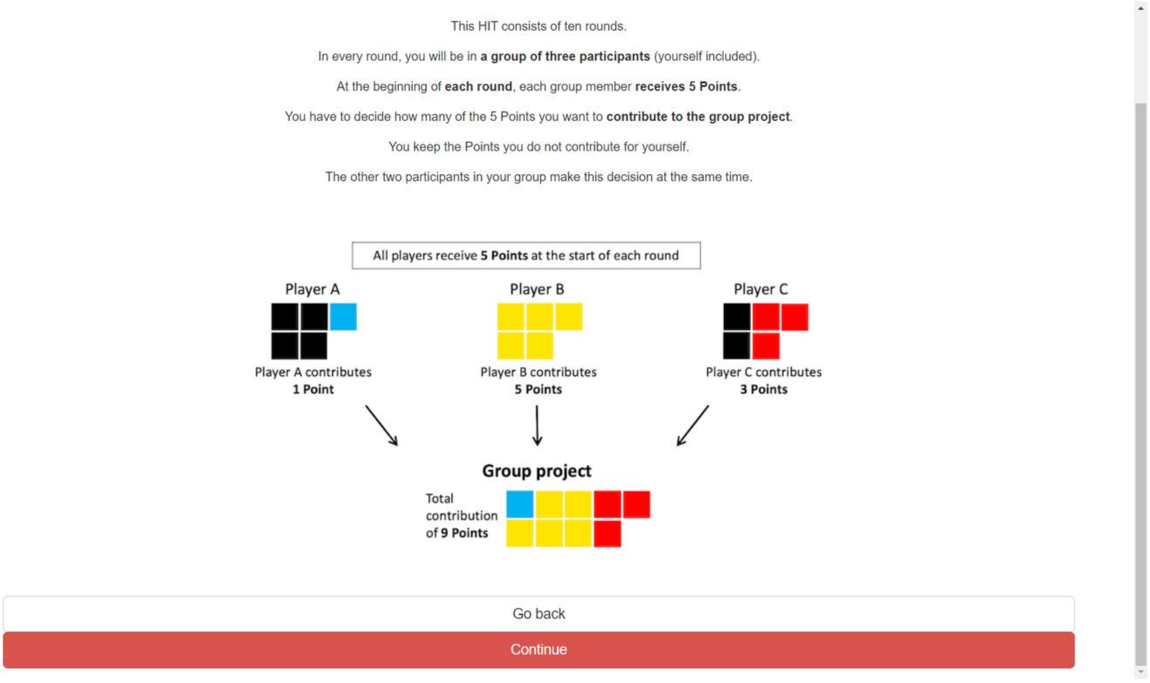


**Fig S2. Instructions screen 1.**

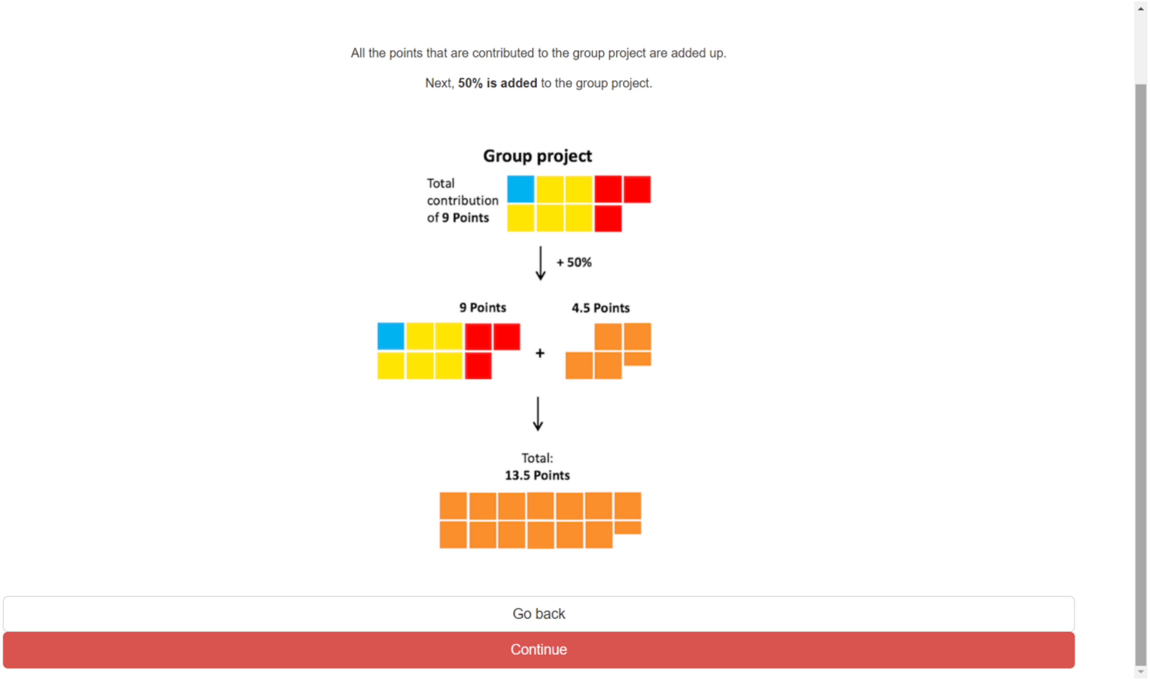


**Fig S3. Instructions screen 2.** For this example screen (and all other example screens), the multiplication factor is 1.5 (MPCR = 0.5). Of course, we did have specifically tailored instructions for each MPCR treatment.

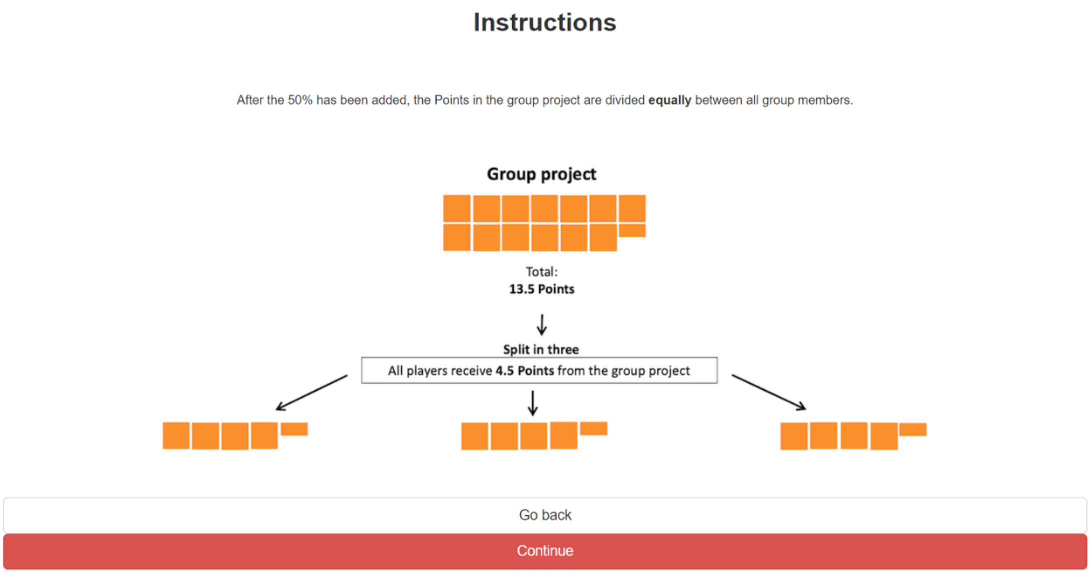


**Fig S4. Instructions screen 3.**

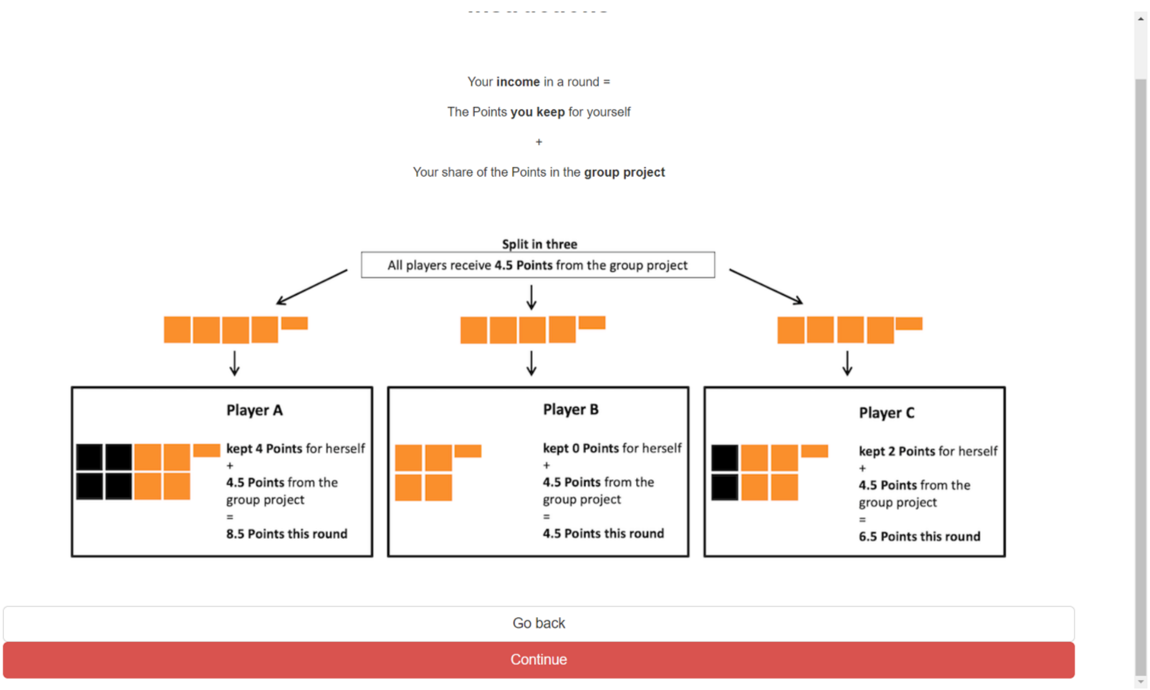


**Fig S5. Instructions screen 4.** The screen after this contained a recap of the entire Public Goods Game logic, including introduction screens 1-4 in a single figure (not shown).

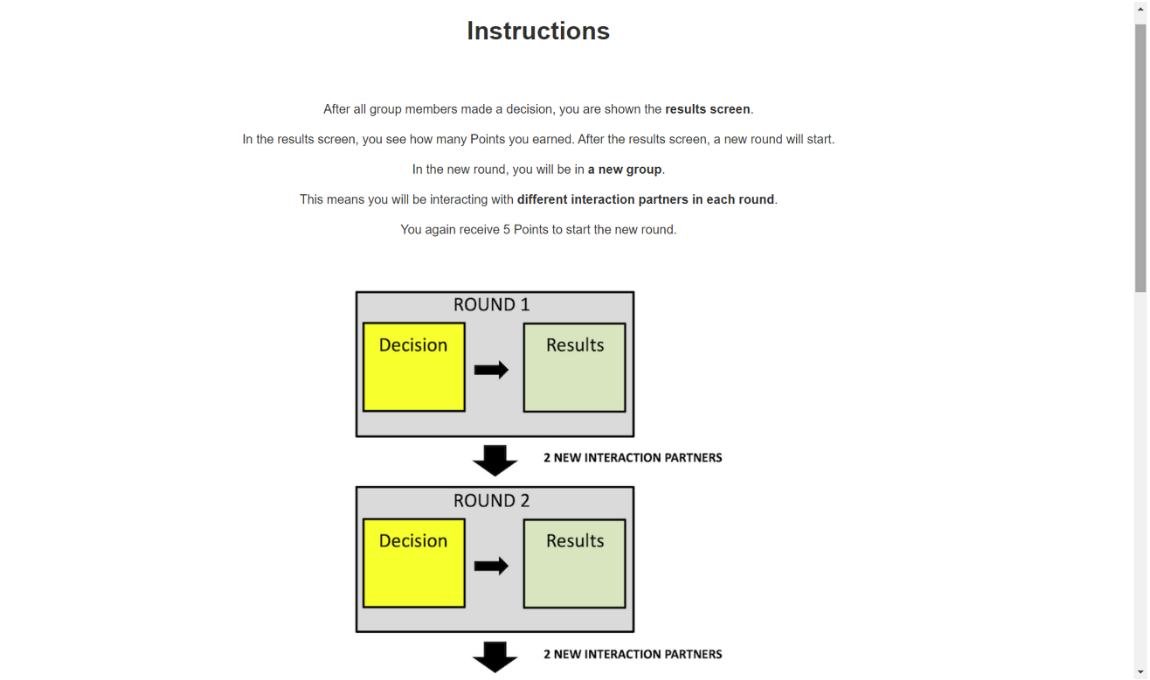


**Fig S6. Top part of Instructions screen 6.**

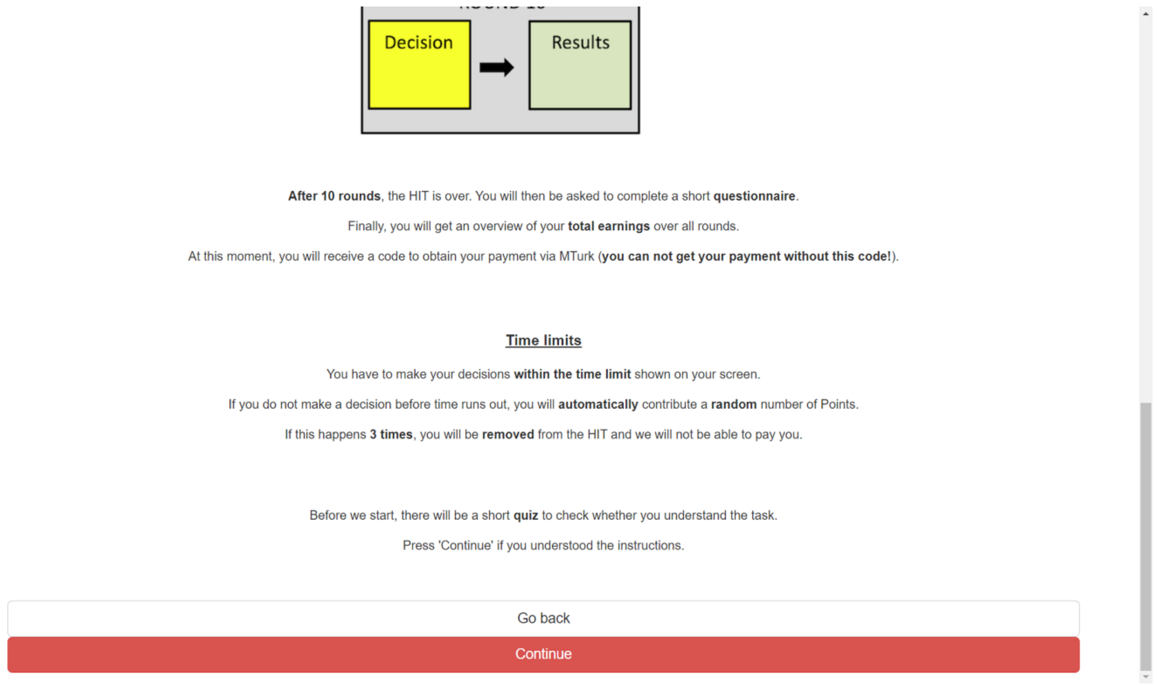


**Fig S7. Bottom part of Instructions screen 6.**

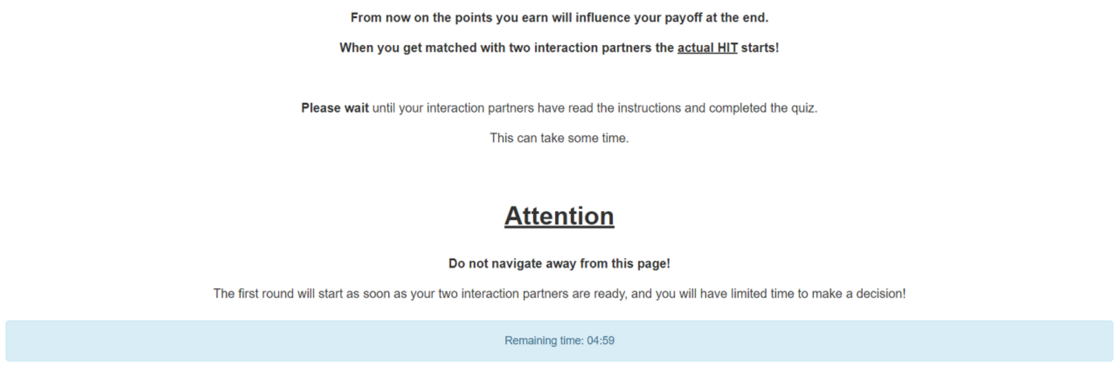


**Fig S8. Lobby screen.** Participants saw this screen while waiting for the game to start.

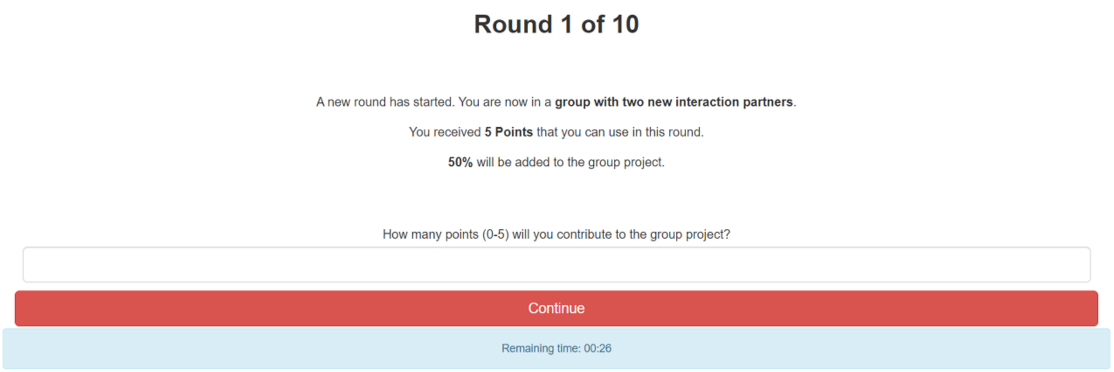


**Fig S9. Decision screen.**

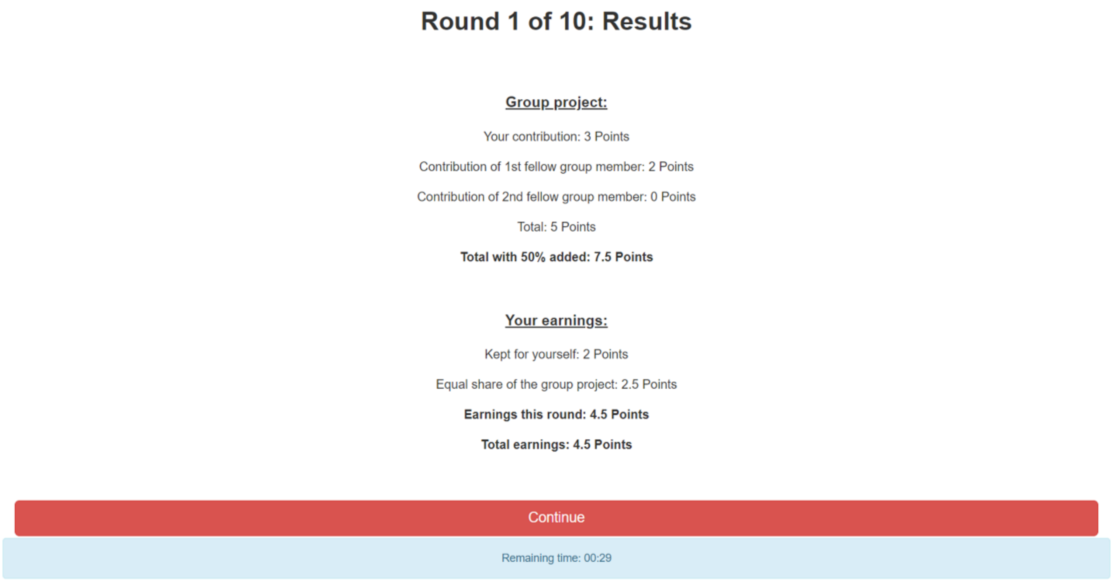


**Fig S10. Results screen.**

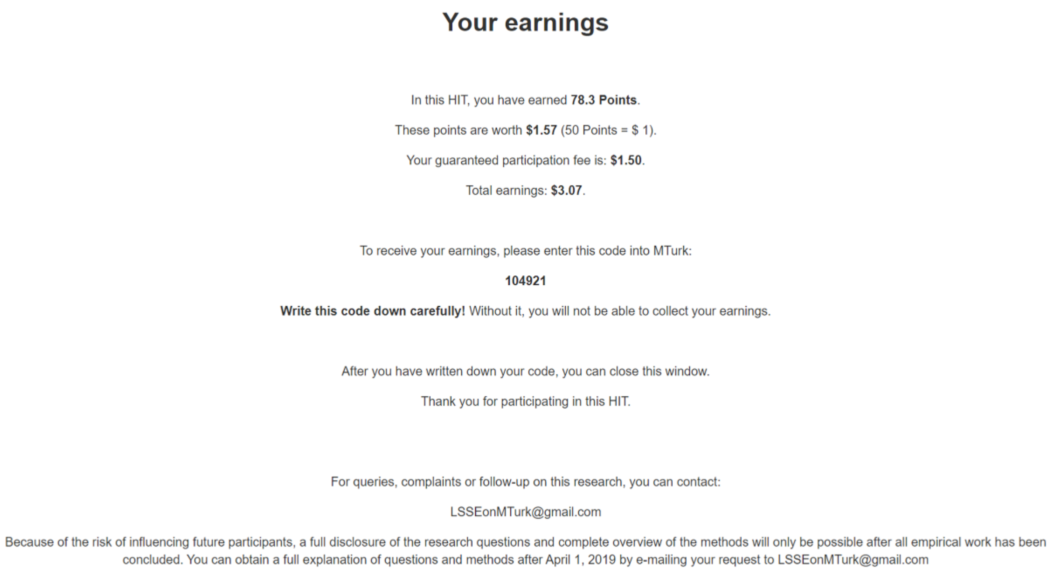
## Your earnings

In this HIT, you have earned **78.3 Points**.

These points are worth **$1.57** (50 Points = $ 1).

Your guaranteed participation fee is: **$1.50**.

Total earnings: **$3.07**.

To receive your earnings, please enter this code into MTurk:

**104921**

**Write this code down carefully!** Without it, you will not be able to collect your earnings.

After you have written down your code, you can close this window.

Thank you for participating in this HIT.

For queries, complaints or follow-up on this research, you can contact:

LSSEonMTurk@gmail.com

Because of the risk of influencing future participants, a full disclosure of the research questions and complete overview of the methods will only be possible after all empirical work has been concluded. You can obtain a full explanation of questions and methods after April 1, 2019 by e-mailing your request to LSSEonMTurk@gmail.com

**Fig S11. Final earnings screen.**

## 2. Comprehension questions to test participants

To make sure participants understood the instructions, there was a page with four multiple-choice comprehension questions after the participants finished all instruction pages. Once they had answered all questions, participants could 'submit' the form. If there were any mistakes, the participants would be explicitly shown which questions they answered incorrectly (without being shown what the right answers were), at which point they could change their answers and could submit again. Participants could only continue in the experiment once they had answered all comprehension questions correctly. If they submitted the form wrongly 10 times, they could not continue with the experiment. The questions (and possible responses) were formulated as follows (bold answers are correct):

Q1. How are the points of the group project divided between the three group members?

A. Group members who contributed more to the group project receive more Points
**B. All group members receive the same number of Points from the group project**
C. The Points from the group project are randomly divided between all group members
D. Group members who contributed less to the group project receive more Points

Q2. True or false? If you contribute more than 0 Points to the group project, your earnings from that round will be more than 5 Points

A. True
B. False
**C. It depends on how many Points the other group members contributed**

Q3. Consider the following two situations:

1. All three group members contributed 0 Points.
2. All three group members contributed 3 Points.

In which situation do you earn more points?

A. In situation 1
**B. In situation 2**
C. I would earn the same number of Points in both situations

Q4. Consider the following two situations:

1. Both your fellow group members contributed 3 Points, but you contributed 0 Points.
2. All three group members contributed 3 Points.

In which situation do you earn more points?

**A. In situation 1**
B. In situation 2
C. I would earn the same number of Points in both situations

44.8% of participants answered all questions correctly on their first try; 84.2% in at most three tries; 94.1% in at most five tries; 0.1% failed to answer the questions correctly within 10 tries. The number of quiz fails significantly predicted contributions, with a higher number of fails being associated with higher contributions (linear mixed regression with 'groupID' as random factor and only 'number of quiz fails' as a predictor; estimate: $0.156 \pm 0.038$, $t = 4.096$, $P < 0.001$).

Although shifted downwards overall, the overall pattern of contributions in response to MPCR remains the same when only participants that finished the quiz in a single try (so without mistakes) are considered (see Figure S12 below). We still observe that participants respond to higher MPCR with higher contributions, and that this pattern is strongest when MPCR are relatively low, leveling off for higher MPCR (around a contribution of 0.55; this was 0.63 if all participants are included). For the data with only participants who made no mistakes in the comprehension test, our model predicts that 80% of the variability in contribution response to MPCR occurs for MPCR below 0.55 (0.58 for all participants), and that 90% occurs for MPCR below 0.60 (0.64 for all participants).

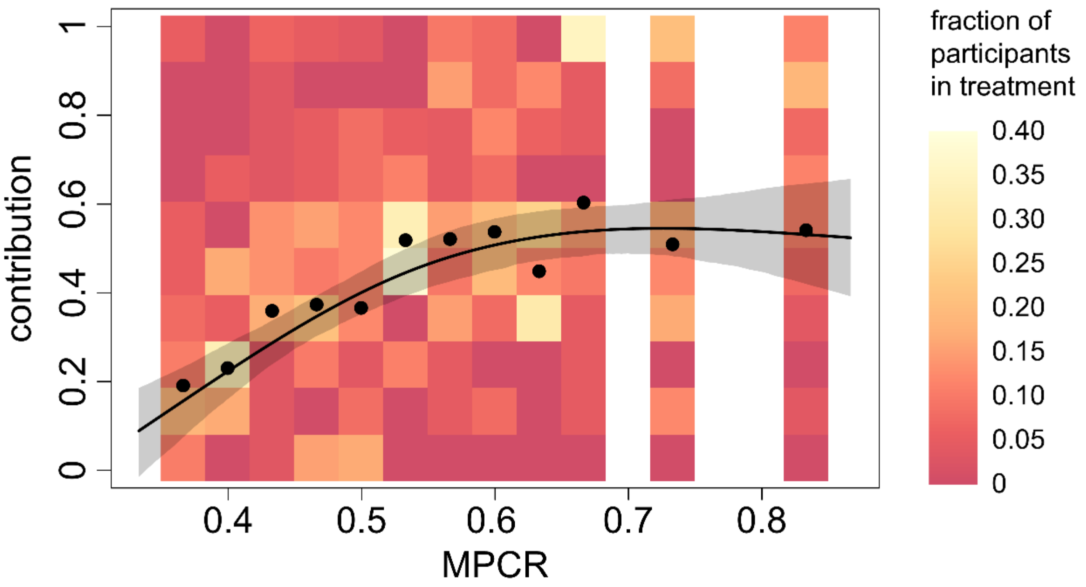


**Fig S12. Cooperation response to MPCR for only participants who made zero mistakes in the comprehension quiz.** Coloured squares indicate the fraction of individuals with an average contribution falling in the indicated range for each treatment (see legend). Black dots show average contribution rates for each treatment. The line shows the contribution response to MPCR predicted by our model based on the data (based on period 6). The shading around the line indicates the 95% confidence interval of the mean.

## 3. Details of statistical models

Our main text presents the results of two statistical models that both infer the effect of MPCR on contributions. One of these models is based on all data (see Fig 1a in the main text), whereas the other infers this relationship for the first rounds only (see Fig 1b). Figure S13 shows effect plots for the former model, visualizing the effects of MPCR and the period (*i.e.*, round) and their interaction on contributions. Fig S15 shows that the decline of contributions over rounds was more pronounced when MPCR was lower than when MPCR was higher. Tables S1 and S2 present the coefficients of this model as well as an Anova-table that specifies the significance of all effects.

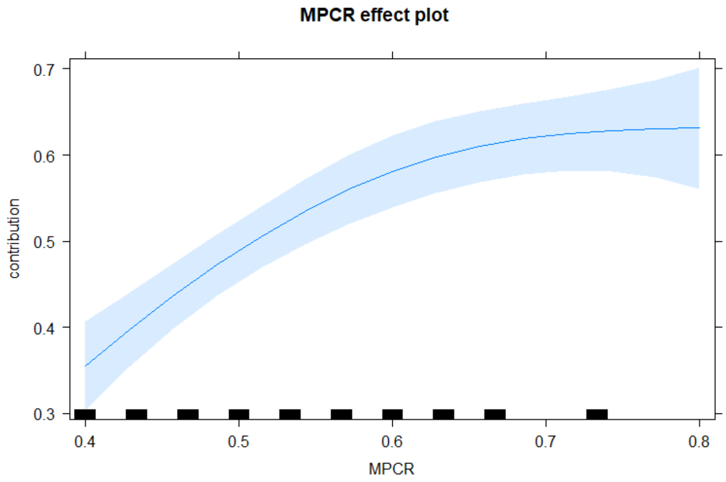


**Fig S13. Effect plot of MPCR for the statistical model described in the Methods section of the main text, which includes all interaction rounds (see Fig 1a).**

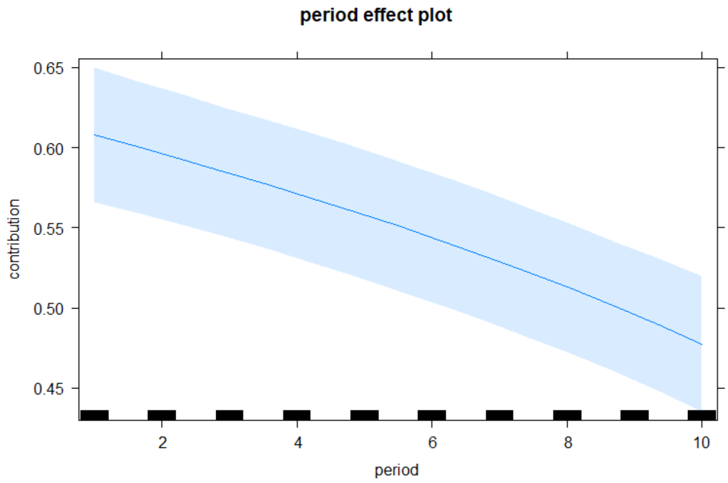


**Fig S14. Effect plot of period for the statistical model described in the Methods section of the main text, which includes all interaction rounds (see Fig 1a).**

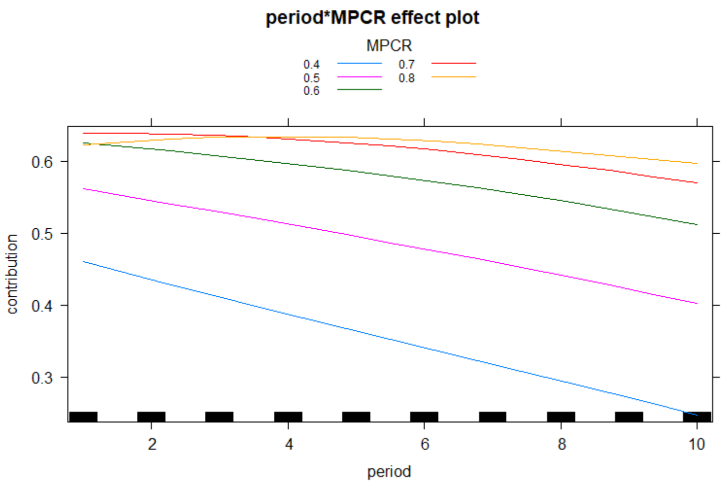


**Fig S15. Effect plot of the interaction of MPCR and period for the statistical model described in the Methods section of the main text, which includes all interaction rounds (see Fig 1a).**

**Table S1. Coefficients of the statistical model described in the Methods section of the main text, which includes all interaction rounds (see Fig 1a).** This model was a mixed-effects model that included individual (“ID”) nested in experimental session (“sessID”) as random effects (see ‘Random effects’), and included natural cubic splines (ns) with two degrees of freedom in function of MPCR, period (*i.e.*, round) and the interaction between both. Note that the estimates and significance of the separate spline terms are given for completeness but not straightforward to interpret – the significance terms of the Anova table (Table S2) gives more direct information about the significance of the effects of the different variables.

```
Random effects:
 Groups     Name        Variance Std.Dev.
 ID:groupID (Intercept) 0.072149 0.26861
 groupID    (Intercept) 0.002815 0.05306
 Residual               0.054381 0.23320
Number of obs: 5764, groups:  ID:groupID, 645; groupID, 36

Fixed effects:
                           Est      StdErr         df t value Pr(>|t|)
(Intercept)            0.42228     0.03570   43.71176  11.830 3.23e-15 ***
ns(MPCR,df=2)1         0.33934     0.08989   59.14345   3.775 0.000373 ***
ns(MPCR,df=2)2         0.05625     0.05638   52.16318   0.998 0.323038
ns(period,df=2)1      -0.55365     0.13123 5155.57496  -4.219 2.50e-05 ***
ns(period,df=2)2      -0.37125     0.06407 5159.34911  -5.795 7.25e-09 ***
ns(period*MPCR,df=2)1  0.58160     0.16990 5155.76589   3.423 0.000624 ***
ns(period*MPCR,df=2)2  0.39750     0.07747 5164.19528   5.131 2.98e-07 ***
---
Signif. codes:  0 ‘***’ 0.001 ‘**’ 0.01 ‘*’ 0.05 ‘.’ 0.1 ‘ ’ 1
```

**Table S2. The outcome of a Type III Anova on the model described in the Methods section of the main text, which includes all interaction rounds (Fig 1a).** This tests for the presence of an effect of each of the fixed predictors after the other predictors in the model.

```
                                Chisq Df Pr(>Chisq)
(Intercept)                   139.954  1  < 2.2e-16 ***
ns(MPCR, df = 2)               14.331  2  0.0007727 ***
ns(period, df = 2)             62.504  2  2.676e-14 ***
ns(period * MPCR, df = 2)      41.586  2  9.326e-10 ***
 ---
Signif. codes:  0 ‘***’ 0.001 ‘**’ 0.01 ‘*’ 0.05 ‘.’ 0.1 ‘ ’ 1
```

Figure S14, Table S3 and Table S4 below respectively show the effects plot, the coefficients of the statistical model, and the Anova table for the model that only takes the first interaction round into account.

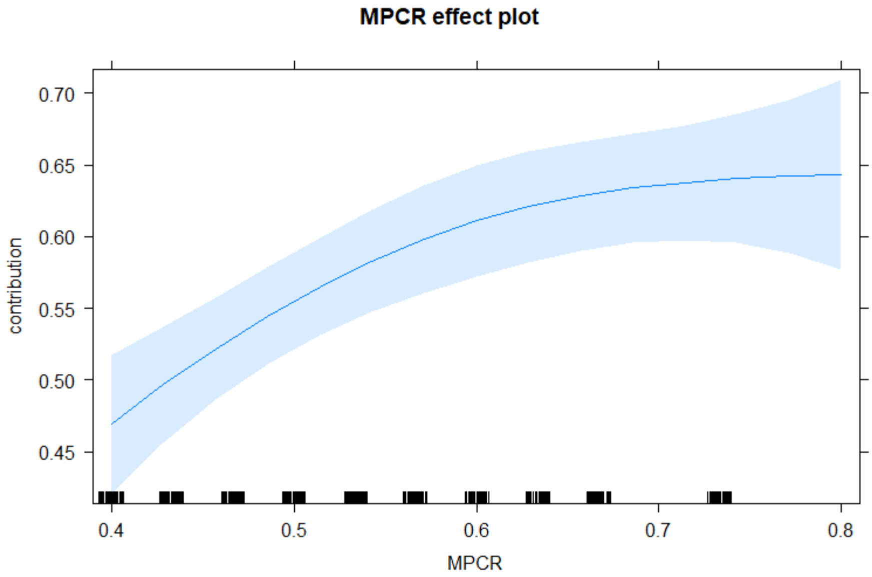


**Fig S16. Effects plot of the statistical model described in the Methods section of the main text, which includes only first interaction rounds (see Fig 1b).**

**Table S3. Coefficients of the statistical model described in the Methods section of the main text, which includes only first interaction rounds (see Fig 1b).** This was a linear model including only the natural cubic spline in function of MPCR (with two degrees of freedom) as a predictor. Note that the estimates and significance of the separate spline terms are given for completeness but not straightforward to interpret – the significance terms of the Anova table (Table S4) gives more direct information about the significance of the effects of the different variables.

```
Coefficients:
                  Estimate Std. Error t value Pr(>|t|)    
(Intercept)        0.43772    0.03184  13.747  < 2e-16 ***
ns(MPCR,df=2)1     0.33329    0.07405   4.501 8.08e-06 ***
ns(MPCR,df=2)2     0.12775    0.04729   2.702  0.00709 ** 

Signif. codes:  0 ‘***’ 0.001 ‘**’ 0.01 ‘*’ 0.05 ‘.’ 0.1 ‘ ’ 1
```

**Table S4. The outcome of a Type III Anova on the model described in the Methods section of the main text, which includes only first interaction rounds (see Fig 1b).** This tests for the presence of an effect of the fixed predictors (in this case, MPCR and the model intercept) after the other predictors in the model.

```
                 Sum Sq  Df F value    Pr(>F)    
(Intercept)      21.478   1 188.984 < 2.2e-16 ***
ns(MPCR,df=2)     2.795   2  12.298 5.787e-06 ***
Residuals        70.349 619                      

Signif. codes:  0 ‘***’ 0.001 ‘**’ 0.01 ‘*’ 0.05 ‘.’ 0.1 ‘ ’ 1
```

## 4. Raw data of contributions over time for each treatment

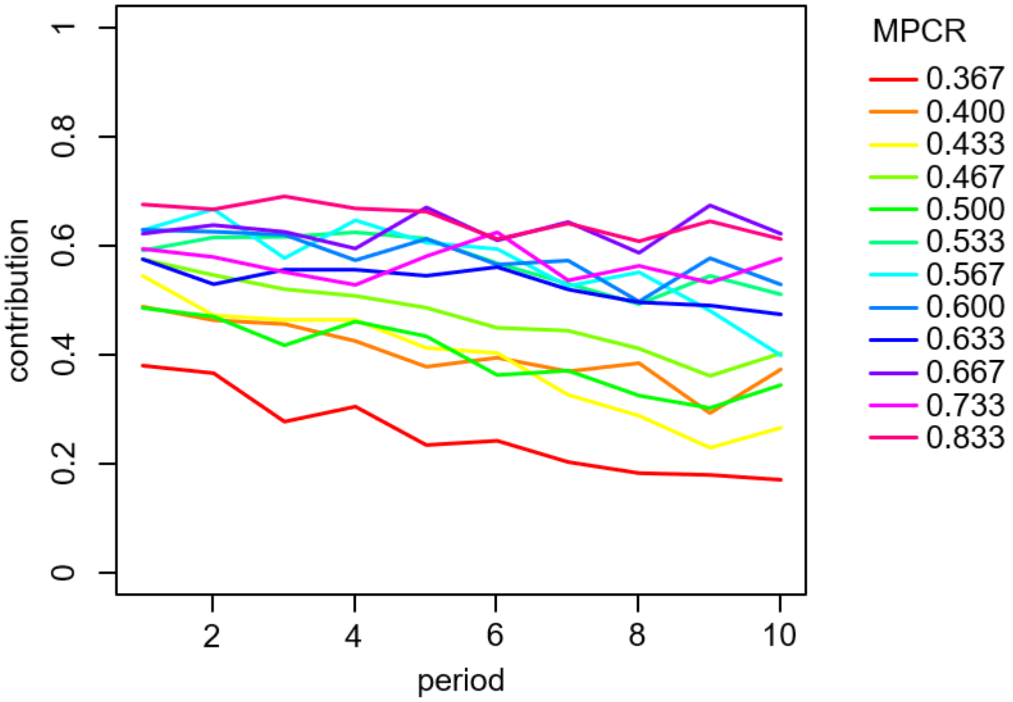


**Fig S17. Average contribution rates over time for each MPCR treatment**. Different colours correspond with the different treatments – see legend.

## 5. The effects of history

Even though the participants in our experiment played a series of one-shot PGGs, it is still possible that outcomes of previous periods affect behaviour in the current period, and this history may affect participants differently depending on the value of MPCR. Here, we investigate this possibility by including a natural cubic spline in function of the average contribution of both interaction partners in the previous interaction round as a variable in the model (adding this variable to the model based on all data described in section 3 of the Supplementary Information). In addition, we include the interaction of this variable with the MPCR treatment. For this model, we did not include data from the first interaction round (because there is no previous round for first rounds). Then, we systematically added and reduced the degrees of freedom of all the spline predictors in the model until we obtained the model with the best AIC. This yielded the model presented below in Figure S18 (effect plots), Table S5 (model estimates) and Table S6 (Anova table).

As can be seen in Fig S18, the models predict that individuals tend to contribute more when their interaction partners contributed more in the previous round. This effect does not meaningfully interact with the MPCR treatment (see also Table S6 below). Indeed, a model that did not include this interaction had a better AIC than the model presented below.

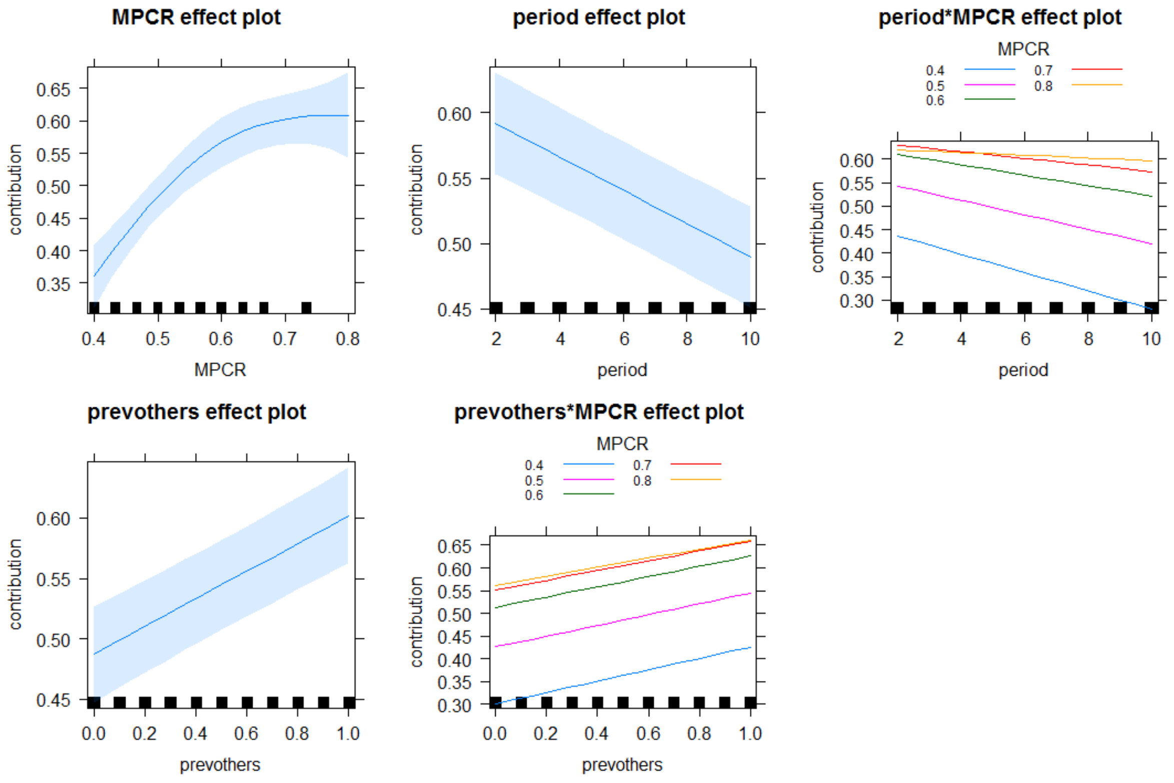


**Fig S18. Effect of MPCR, period, and previous average contributions by interaction partners on contributions.** Variable 'prevothers' indicates the effect of the average contribution by the interaction partners in the previous round.

**Table S5. Coefficients of the statistical model including the effects of history.** This model was a mixed-effects model that included individual ("ID") nested in experimental session ("sessID") as random effects (see 'Random effects'), and included natural cubic splines (ns) in function of MPCR (with two degrees of freedom), period (*i.e.*, round; one d.f.), the interaction between both (one d.f.), the average contribution of both interaction partners in the previous round ('prevothers', one d.f.), and the interaction of that variable with MPCR (one d.f.). Note that the estimates and significance of the separate spline terms are given for completeness but not straightforward to interpret – the significance terms of the Anova table (Table S2) gives more direct information about the significance of the effects of the different variables. A model that did not include the interaction of the variables 'prevothers' and 'MPCR' yielded better AIC.

```
Random effects:
 Groups     Name        Variance Std.Dev.
 ID:groupID (Intercept) 0.07579  0.27529
 groupID    (Intercept) 0.00169  0.04111
 Residual               0.05268  0.22953
Number of obs: 5142, groups:  ID:groupID, 639; groupID, 36

Fixed effects:
                         Estimate  StdErr         df t value Pr(>|t|)
(Intercept)               0.33707 0.03365   43.42213  10.017 7.36e-13 ***
ns(MPCR,df=2)1            0.37648 0.08136   49.93990   4.628 2.66e-05 ***
ns(MPCR,df=2)2            0.08611 0.05578   62.80646   1.544  0.12767
ns(period,df=1)          -0.36153 0.05525 4538.10766  -6.543 6.70e-11 ***
ns(period*MPCR,df=1)      0.39545 0.08984 4535.48382   4.402 1.10e-05 ***
ns(prevothers,df=1)       0.18579 0.06905 4623.63610   2.691  0.00715 **
ns(prevothers*MPCR,df=1) -0.06224 0.09632 4621.39125  -0.646  0.51819

Signif. codes:  0 '***' 0.001 '**' 0.01 '*' 0.05 '.' 0.1 ' ' 1
```

**Table S6. The outcome of a Type III Anova on the model including the effects of history.** This tests for the presence of an effect of each of the fixed predictors after the other predictors in the model.

```
                            Chisq Df Pr(>Chisq)
(Intercept)              100.3325  1  < 2.2e-16 ***
ns(MPCR,df=2)             21.4157  2  2.237e-05 ***
ns(period,df=1)           42.8111  1  6.029e-11 ***
ns(period*MPCR,df=1)      19.3767  1  1.073e-05 ***
ns(prevothers,df=1)        7.2407  1   0.007127 **
ns(prevothers*MPCR,df=1)   0.4175  1   0.518162
---
Signif. codes:  0 '***' 0.001 '**' 0.01 '*' 0.05 '.' 0.1 ' ' 1
```
